# Acupuncture combined with Tuina in the treatment of cervical longus tendinitis: A case report

**DOI:** 10.1097/MD.0000000000035980

**Published:** 2023-11-17

**Authors:** Meng Guo, Min Zhang, Xiaole Guo, Hongfeng Wang, Hui Li

**Affiliations:** a Department of Acupuncture and Tuina, Changchun University of Chinese Medicine, Changchun, China; b Changchun University of Chinese Medicine, Changchun, China; c Department of Anorectal, The First Affiliated Hospital, Changchun University School of Medicine, Changchun, China.

**Keywords:** acupuncture, case report, tendinitis of the cervical longus muscle, Tuina

## Abstract

**Introduction:**

Cervical longus tendonitis is a type of disease with neck pain as the main clinical manifestation. Because the front of the cervical longus muscle is adjacent to the esophagus and pharynx, this disease is often accompanied by pharyngeal pain and pain when swallowing. Clinical and imaging doctors often have an incomplete understanding of it, and this disease is often confused with other diseases that cause neck pain.

**Patient concerns:**

A 33-year-old Chinese woman was the patient. Suffering from severe neck pain and significantly limited activity, accompanied by left shoulder pain, occasionally dizziness, headache and other symptoms, the pain is significantly aggravated when doing swallowing action.

**Diagnosis:**

Tendonitis of the long neck muscle.

**Interventions:**

Given the patient’s condition, we used acupuncture combined with massage therapy as a symptomatic treatment.

**Outcomes:**

After 10 days of treatment, the symptoms were better than before, and no pain was seen in the swallowing movements such as drinking water (Fig. 2C and D).

**Lessons:**

Because the clinical reports of diseases are rare, the treatment methods are limited, and acupuncture combined with massage is an effective method for the treatment of tendonitis of the cervical long muscle, to dredge the meridians, promoting blood circulation, removing blood stasis and relieving pain.

## 1. Introduction

Cervical longus tendonitis is a type of disease with neck pain as the main clinical manifestation. Analysis of the patient’s clinical symptoms, with acute neck pain, neck stiffness, and dysphagia of the cervical chronic muscle tendinitis the typical triad.^[1]^ The cause of cervical long muscle tendinitis is associated with a series of symptoms^[2]^ caused by the deposition of hydroxyapatite crystals on the upper oblique muscle of the cervical long muscle, causing a local inflammatory response to stimulate local tissue exudation. The mechanism of crystalline deposition of hydroxyapatite is not well defined and may be related to factors such as injury, tissue necrosis, and inflammation. The most common form of clinical cervical long muscle tendonitis is characterized by acute or subacute neck pain, limited mobility and dysphagia, severe neck pain during continuous movement, a significantly limited range of motion, an increased pain during swallowing movements, and difficulty in drinking and eating, which affects the daily life of patients. Patients with cervical tendonitis occasionally have fever, with leukocytosis, an increased erythrocyte sedimentation rate and C-reactive protein.

At present, due to the rare clinical reports of the disease, the treatment methods are relatively limited, and acupuncture combined with massage is an effective method for the treatment of cervical long muscle tendinitis, to dredge the meridians, promote blood circulation, remove blood stasis, and relieve pain.

## 2. Case report

### 2.1. Clinical presentation

**Figure 1.**
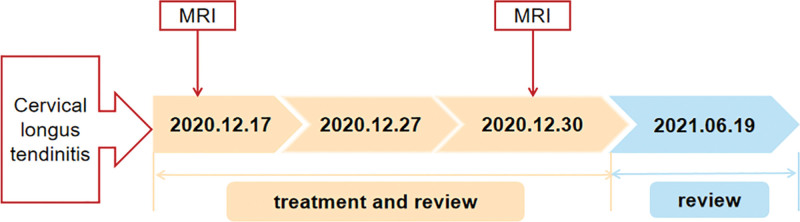
Timeline of clinical and procedural data.

The timeline with clinical and procedural data is shown in Figure 1.

A 33-year-old woman with a tendinitis injury. The patient arrived at the Acupuncture Tuina Center with a complaint of severe neck pain. The movement of the neck was obviously limited in the morning, and was accompanied by left shoulder pain, occasional dizziness, a headache, and other symptoms. Moreover, the pain was significantly aggravated when swallowing.

*History of past illness*: The patient bowed her head and worked at her desk for a long time, with neck blood circulation disorders. One day ago, she showed neck pain, accompanied by left shoulder pain, and mistook it for cervical spondylosis. Personal and family history: The patient denied family history of similar disease.

**Figure 2.**
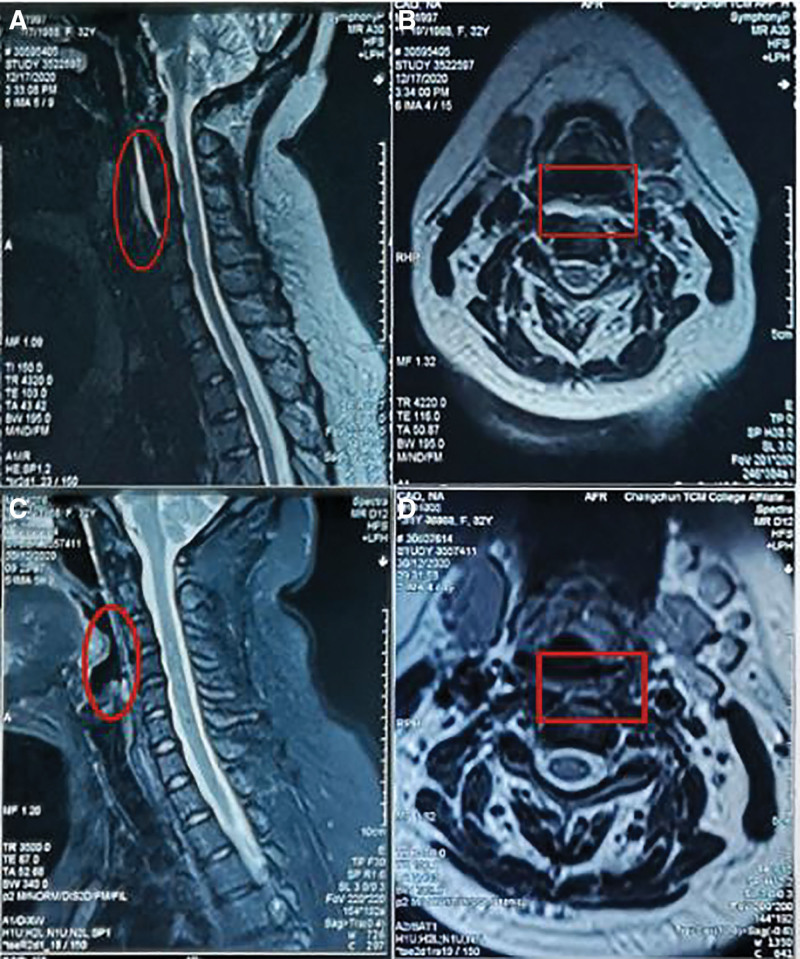
MRI of the cervical spine before acupuncture treatment: (A) cervical spine MRI (sagittal position); (B) cervical MRI (cross section). MRI of the cervical spine after acupuncture treatment: (C) cervical spine MRI (sagittal position); (d) cervical MRI (cross section). MRI = magnetic resonance imaging.

*Physical examination*: Body temperature, 36.5°C; blood pressure, 124/80 mm Hg; heart rate, 90 beats per min; respiratory rate, 18 breaths per min. Furthermore, due to the patient’s severe pain, I was unable to check the cervical mobility, neck 3 to 7 transverse process tenderness (+), neck 3-thoracic 1 spine process and bilateral open 1.0 cm tenderness (+), percussion pain (+), left foraminal extrusion test (+), left brachial plexus pull test (+), bilateral biceps, triceps tendon reflex, bilateral homann sign (−). Biochemical examination showed leukocytosis, an increased erythrocyte sedimentation rate, and C-reactive protein (a white blood cell count was performed in 433.60/µL; the erythrocyte sedimentation rate was 30mm/h; with a C-reactive protein value of 5.32 mg/dL). X-ray of the open side of the cervical spine: the atlantoaxial joint space is acceptable, the physiological curvature of the cervical spine is straight, and the foramina of C3-4 and C4-5 becomes smaller. MRI of the cervical spine (Fig. 2A and B): the cervical vertebrae are arranged neatly, the physiological curvature is straight, the signal of the cervical discs decreases in T2WI, the cervical discs 4/5 and 6/7 discs protrude backward, and the dural sac is compressed; see the strip long T2 high signal in the anterior cervical space. As a result, the patient was diagnosed as having cervical long muscle tendonitis.

### 2.2. Interventional procedure

Acupuncture combined with massage therapy was used. Acupuncture and moxibustion: Shentin, Baihui, Chenjiang, Tianzhu (double), Yongquan (double). Specification of the needle selection: 0.25mm * 50mm (Guizhou Andy Pharmaceutical Equipment Co., LTD., standard no.: GB2024-94). Needle method: Shangxin 1-1.3 inches, upward oblique stab 0.3-0.5 inch, Tianzhu point straight stab 0.5-0.8 inch, Yongquan point straight stab 0.5 inches. After applying the method to get gas, leave the needle for 30 min. The starting and ending point of the long neck muscle is selected: 0.25mm * 50 mm (Guizhou Andy Pharmaceutical Equipment Co., LTD., standard no.: GB2024-94). Needle method: first find the start and end point of the neck long muscle, directly stab the needle 0.5-1 inch at the starting point and stop position, twist with light finger force, do not insert the needle, until the patient feels sore swelling, immediately release the needle, taking the needle. At the same time, combine cervical IF pulse electrical therapy; on the second day, the pain is relieved during drinking and eating. On the third day, for the projection point of the long cervical muscle surface, massage was used for treatment, mainly for local relaxation. After 3 days of treatment, the patient reported that the neck pain was significantly improved, and the pain was significantly reduced when swallowing. Now, it mainly showed posterior occipital pain, an occasional headache and dizziness. Combined with the patient’s symptoms, massage combined with atlantoaxial directional inverted reduction technique was given. The specific operations are as follows: the patient is instructed to lie in the supine position, and the operator sits in front of the head, first by applying the massage method of pressing and rubbing, elastic and pulling out, and focus on the rectus of the head, the oblique and the occipital atlantoaxial. Then the manual reduction of the atlantoaxial joint: the patient’s head is rotated clockwise to a slight resistance, the middle finger of the left hand is pulled to the left, the right palm supports the jaw, and the head is slowly raised to the maximum, the patient’s whole body is relaxed instantly, and to the right along the crown axis. The patient’s head was rotated counterclockwise to a slight resistance, the right thumb pushed the vertebral spine to the left, the left palm supported the jaw, and slowly raised the head to the maximum, the patient’s whole body relaxed instantly, and the left hand quickly touched the head along the coronal axis. Then to take the method, press and knead techniques, relax the neck pillow, to the head after the rectus muscle, head oblique muscle, head lower oblique muscle as the focus.

### 2.3. Outcome and follow-up

On 19 June 2021, 6 months after therapy concluded, the patient was contacted via telephone. Since then, the patient has had no more treatment, and the therapeutic benefits of acupuncture combined with massage therapy have remained excellent, with no return of clinical problems. MRI result report (Fig. 2C and D): On examination, the cervical vertebrae were arranged neatly, the physiological curvature becomes straight, the leading edge of the C3-7 vertebra became pointed, the signal of the cervical disc in T2WI decreased, the C4/5 and 6/7 discs expanded backward, and the dural sac was compressed, the cervical cord left shape, thickness and signal as usual, no occupying space in the spinal canal, and no abnormal signal was found in the paravertebral soft tissue.

## 3. Discussion

In the authors’ opinion, in Chinese journals the published literature is small, and most of this is published by imaging doctors, which has certain limitations with respect to clinical understanding. Langford et al,^[3]^ in a report of a patient with severe neck pain and dysphagia, due to the rarity of cervical tendon tendinitis, the initial misdiagnosis of patients as having a retropharyngeal abscess before establishing the correct diagnosis of acute calcified cervical tendon tendinitis. In addition, in 26 clinical symptoms of,^[4]^ “10 patients had shoulder and arm pain, 5 patients had pain swallowing, the duration of symptoms was 1 to 3 months, and 5 patients had a history of minor trauma 2 days before the onset. All patients had no fever, and 8 cases had erythrocyte sedimentation and increased C-reactive protein; however, white blood cell counts were normal, and 18 patients had calcification deposition below the anterior arch of the atlas and the anterior front of the pivotal vertebra, which is the attachment point of the cervical long muscle; 23 patients had prevertebral effusion at cervical level 1 to cervical 4; 18 patient had symptoms that disappeared within 1 week after onset, and 8 subacute patients recovered 6 weeks after onset.” According to a review of the literature by Park et al,^[5]^ the most common symptoms were neck pain (94%), limited range of motion (45%), odynophagia (45%), neck stiffness (42%), dysphagia (27%), sore throat (17%), and neck spasms (11%). Therefore, some scholars believe that cervical long muscle tendinitis is a typical triad of acute neck pain, neck stiffness, and dysphagia, often accompanied by limited cervical movement, a sore throat, pharyngeal edema, a posterior occipital headache, torticollis, dizziness, and increased inflammatory indicators, with an average annual incidence of 0.50/100,000 person-year.^[6]^ Also, the age of the patient must be a factor for consideration as tendonitis is described only in the adult population. Therefore, when pediatric patients have similar clinical manifestations, a cervical deep space infection should be suspected, such as retropharyngeal or parapharyngeal abscess.^[7]^

RI and CT are also important adjuncts to identify tendonitis. MRI is the primary means of diagnosing this disease. MRI examination T2 weighted localized fissure-like areas in the prevertebrae, mostly located at the level of cervical 1-cervical 4, with high signal changes, and this response is due to effusion caused by inflammation. T2 weighting, fat suppression sequence, and T1 weighting have important roles in identifying effusion and adipose tissue, adipose-containing bone marrow, as well as retropharyngeal infection, and spondylitis.^[8]^ CT examination is performed by high resolution to identify calcification within the tendon, which is often not able to be seen in plain radiographs and is able to define calcification rather than a high-density shadow of other skeletal sources. Calcification is generally located below the anterior arch and ahead of the pivot dentate process. CT helps to clarify the presence of prevertebral effusion and to exclude other pathological findings such as fracture or abscess. When the soft tissue swelling of the anterior edge of the C1-4 vertebral body and effusion coexist with irregular calcification below the anterior arch of the C1 vertebral body, it is helpful in the diagnosis of tendonitis.^[4]^ It is therefore concluded that the imaging findings specific to tendonitis of the cervical muscle are the coexistence of the anterior rim effusion of the cervical 1 to cervical 4 vertebrae with the calcification deposition below the anterior arch of the cervical 1 vertebra. The most common clinical manifestations are acute and subacute episodes of neck pain and tenderness, limited mobility, and dysphagia and increased sedimentation and C-reactive protein.^[9]^ In short, cervical long muscle tendinitis is a self-limiting disease, often treated with conservative treatment, can be completely cured. The combination of clinical findings and imaging findings will help to differentiate this from other diseases.

In terms of treatment, at present most of the oral non-steroidal anti-inflammatory analgesics and neck braking are treated with a conservative approach. Non-steroidal anti-inflammatory drugs are commonly used in clinical practice, and these are the same drugs widely used to treat various inflammatory diseases. However, many researchers have realized that non-steroidal anti-inflammatory drugs may cause adverse reactions and cardiovascular events in the digestive tract and even threaten the life of patients in serious cases.^[9]^ The treatment of tendonitis by taking non-steroidal anti-inflammatory analgesics has certain side effects, and antibiotics, topical analgesic therapy, extracorporeal shock wave therapy, and surgery are not recommended.^[10]^ According to the authors’ literature research, there is no other treatment method for this disease except oral medicine. The Chinese acupuncture and massage proposed in this paper combines physical therapy to treat cervical longus muscle tendonitis, and this can stimulate acupuncture points to reconcile the body qi, blood and Yin and Yang, cooperate with physical therapy, and have the effect of relaxing tendons and activating collaterals, removing blood stasis, and promoting blood circulation. The combination of traditional Chinese acupuncture and massage and physical therapy can better relieve the local pain of patients with cervical long muscle tendonitis, reduce the limitation of neck movement, and improve the cervical spine function of patients. This treatment is inexpensive, with high degree of safety, no side effects, good patient acceptance, and good clinical efficacy, which warrants more promotion and additional research.

## 4. Conclusion

In conclusion, this case provides solid clinical evidence for the use of acupuncture combined with Tuina in the treatment of cervical longus tendonitis, especially for cervical longus tendonitis injury, and proves a new strategy for the treatment of cervical longus tendonitis. Compared with other treatment methods, the combination of Chinese acupuncture, massage and physical therapy for cervical long muscle tendonitis is more optimized and more conducive to the patients’ acceptance.

## Acknowledgements

The authors would like to thank the patient and his guardians.

## Author contributions

**Conceptualization:** Min Zhang.

**Methodology:** Meng Guo.

**Resources:** Meng Guo, Hui Li.

**Supervision:** Xiaole Guo.

**Writing – original draft:** Meng Guo, Min Zhang.

**Writing – review & editing:** Hongfeng Wang.
